# Effects of taurine on metabolomics of bovine mammary epithelial cells under high temperature conditions

**DOI:** 10.3389/fvets.2024.1393276

**Published:** 2024-06-10

**Authors:** Feifei Liu, Liang Liang, Zonggang Luo, Gongwei Zhang, Fuyuan Zuo, Ling Wang

**Affiliations:** ^1^College of Animal Science and Technology, Southwest University, Rongchang, Chongqing, China; ^2^Beef Cattle Engineering and Technology Research Center of Chongqing, Southwest University, Rongchang, Chongqing, China

**Keywords:** metabolites, cattle, heat stress, metabolic pathways, taurine

## Abstract

High temperature induces heat stress, adversely affecting the growth and lactation performance of cows. Research has shown the protective effect of taurine against hepatotoxicity both *in vivo* and *in vitro*. This study aimed to investigate the effect of taurine on the metabolomics of mammary epithelial cells of dairy cows under high-temperature conditions. Mammary epithelial cells were exposed to 0 mmol/L (HS, control), 8 mmol/L (HT-8), and 32 mmol/L (HT-32) of taurine, then incubated at 42°C for 6 h. Metabolomics analysis was conducted using Liquid Chromatograph Mass Spectrometer (LC–MS). Compared with the HS group, 2,873 and 3,243 metabolites were detected in the HT-8 group in positive and negative ion modes. Among these, 108 and 97 metabolites were significantly upregulated in positive and negative ion modes, while 60 and 166 metabolites were downregulated. Notably, 15 different metabolites such as palmitic acid, adenine and hypoxanthine were screened out in the HT-8 group. Compared with the HS group, 2,873 and 3,243 metabolites were, respectively, detected in the HT-32 group in the positive and negative ion modes. Among those metabolites, 206 metabolites were significantly up-regulated, while 206 metabolites were significantly downregulated in the positive mode. On the other hand, 497 metabolites were significantly upregulated in the negative mode, while 517 metabolites were reported to be downregulated. Noteworthy, 30 distinct metabolites, such as palmitic acid, phytosphingosine, hypoxanthine, nonanoic acid, and octanoic acid, were screened out in the HT-32 group. KEGG enrichment analysis showed that these metabolites were mainly involved in lipid metabolism, purine metabolism and other biological processes. Overall, our study indicates that taurine supplementation alters the metabolites primarily associated with purine metabolism, lipid metabolism and other pathways to alleviate heat stress in bovine mammary epithelial cells.

## 1 Introduction

In livestock production, stress can be induced by factors such as environment, nutrition, management, pathogens and disease (1). Climate change-induced increases in diurnal temperatures and global warming have attracted increasing attention. Heat stress (HS) happens when an animal is not able to dissipate the heat load generated by the body’s metabolism and the environment, thereby losing the body’s thermal homeostasis. The cows under HS conditions reduce the body heat load by reducing dry matter intake (DMI), resulting in insufficient energy and nutrients being available to maintain normal production (2–4). HS reduces daily rumination time and milk production in lactating high-producing dairy cows (5, 6). Furthermore, HS poses a significant threat to dairy farming, disrupting cows’ productivity, reproductive performance, and overall health (7, 8). In addition, animal management has become a huge challenge due to the increase in the number of production animals and the increased metabolic activity due to high temperatures (9). Therefore, HS caused to huge economic loss on dairy production in the world.

The structural and functional integrity of the mammary tissue is considered a crucial factor in the performance of lactation in cows (10). Research has shown that environmental and management factors can affect mammary gland function at both molecular and cellular levels (11). For example, high temperatures can inhibit the proliferation of mammary epithelial cells, causing the occurrence of various diseases such as mastitis, and affecting the yield and milk quality of cows (12–14). HS led to a variety of protein chaperone genes up-regulated and interfere with cytoskeletal and cell transport function in bovine mammary epithelial cells *in vitro* (15).

Taurine, as a sulfur-containing non-protein amino acid, is one of the most abundant free amino acids in mammalian tissues (16). It is involved in many biological processes, including anti-inflammatory, antioxidant activities, bile acid binding, membrane stabilization, osmoregulation, regulation of cellular calcium flux, and immunomodulation (17, 18). It has been shown to be protective in stress models and toxic situations such as high temperatures, endotoxin excitation, and high stocking densities (19). Previous studies have shown that taurine supplementation promotes milk fat and protein synthesis and alleviates oxidative stress and inflammation in bovine mammary epithelial cells (20, 21). Moreover, taurine can reverse the decreases in the activity of superoxide dismutase and glutathione peroxidase induced by HS, then alleviate cellular oxidative stress, and thus protect bovine mammary epithelial cells against HS (22). Furthermore, the protective effect of taurine on mammary glands has been achieved by attenuating mammary gland epithelial integrity damage and inflammatory response under HS conditions (23).

The metabolites of the organism, especially the macromolecules, can directly reflect the changes in life activities in the organism (24). Metabolomics is an important method that allows for a comprehensive analysis of metabolites in the organism (25). It can be used to explore the dynamic response of living system metabolites to changes in endogenous or exogenous factors in a quantitative way (26). Previous research has demonstrated that HS results in an elevated concentration of amino acids in the mammary epithelial cells of dairy cows, thereby enhancing the transportation of amino acids and stimulating the activity of the mTOR signaling pathway (27). Additionally, 16 metabolites have been identified in the plasma of dairy cows treated with N-Carbamylglutamate (NCG), and found that NCG could relieve HS, resulting in an improvement in milk production under HS (28). Furthermore, another study using analyzing metabolomics proved the protective effect in the yeast chromium by reducing rectal temperature, decreasing serum insulin concentration, and increasing serum glucose and plasma nicotinamide concentration to improve the lactation performance of dairy cows under heat stress (29).

It has been found that heat stress in dairy cows is associated with many metabolic pathways, such as glucose metabolism, amino acid metabolism, and nucleotide metabolism (30, 31). However, the direct impact of taurine on the metabolism of bovine mammary epithelial cells under HS remains underexplored. This study aims to address this gap by employing liquid chromatography-mass spectrometry (LC-MS) to investigate the metabolite profile of bovine mammary epithelial cells supplemented with taurine under high-temperature conditions. By shedding light on the molecular mechanisms underlying taurine’s protective effects, this research seeks to provide a theoretical basis for developing targeted interventions to mitigate the adverse effects of HS in dairy cows.

## 2 Materials and methods

### 2.1 Cells and reagents

The bovine mammary epithelial cells were preserved by our laboratory (Rongchang, Chongqing); fetal bovine serum and DMEM/F-12 medium were purchased from Gibco; taurine (Taurine, 99% biotech grade) was purchased from Macklin, and methanol (chromatographically pure, HPLC-grade) were purchased from Sigma.

### 2.2 Cell culture and treatment

The mammary epithelial cells were cultured in DMEM/F-12 medium containing 10% fetal bovine serum and 100 U/mL penicillin and 100 μg/mL streptomycin at 37°C, 5% CO_2_ in an incubator, and then cultured in 10 cm dishes at a density of 6 × 10^6^ cells/mL until cells reached 80–90% confluence, and then replaced with culture medium containing 0 mmol/L (HS, control group), 8 mmol/L (HT-8) and 32 mmol/L (HT-32) of taurine, respectively. After cells were cultured at 37°C for 6 h, they were then transferred to 42°C for 6 h-incubation based on similar work by Salama et al. (32–34). All of the treatments were conducted in six biological replicates (*n* = 6).

### 2.3 Samples research topic

After the hyperthermia treatment, the medium was discarded and washed twice with pre-cooled (4°C) PBS. Afterwards, the dish containing the cells was exposed to liquid nitrogen for 10 s and then placed on ice. 750 μL of methanol–water (Methanol/Water = 8:2, V/V) was added to each dish, and the cells were scraped off with a cell scraper and transferred into Eppendorf tubes, while the remaining cells were scraped off with another 750 μL of methanol–water and put into the same tube. The collected samples were stored at −80°C for 30 min (mins) and then pulverized by ultrasound. The samples were centrifuged at 14,000 g for 15 min at 4°C. The supernatant was transferred in a 1.5 mL Eppendorf tube and stored at −80°C for measurement.

### 2.4 Detection of metabolites

LC-MS/MS analysis was performed using UHPLC equipment (UHPLC3000, Dionex, Sunnyvale, CA, Germany). Detection conditions were presented as follows.

#### 2.4.1 Chromatographic conditions

Column: UPLC Hypersil Gold C18 column (2.1 Hypers, particle size 1.91, Thermo Fisher Science, United States) and Q-Exactive Orbitrapp (Thermo Fisher Science, United States), flow rate 0.2 mL/min, column temperature 35°C, injection volume 2 μL; mobile phases: solvent A (0.1%formic acid), solvent B (methanol containing 0.1% formic acid), solvent C (0.1%NH3), and D (methanol containing 0.1%NH3); gradient elution program: positive ions were 0–10 min, 5%B and 95%A; 10–12 min, 5%A and 95% B; 12–13 min, 5%A and 95%B; 13.1–14 min 95%A and 5%B; negative ions were 0–2.5 min, 95%C and 5%D; 2.5–16.5 min, 95%D and 5%C; 16.5–19 min, 95%D and 5%C; 19–20 min, 95%C and 5%D.

#### 2.4.2 Mass spectrometry conditions

MS/MS spectra were acquired in an information-dependent acquisition (IDA) mode using Q-ExactiveOrbitRAP under the control of the acquisition software (X Cup, Thermo Fisher Science, United States); HASI source operating parameters: sheath and auxiliary gas flow rates of 40 and 10 arb, respectively, capillary temperature of 320°C, full mass scan range m/z 70–1,050 with a resolution of 70,000; the MS/MS scan mode was set to a data-dependent MS2 (dd-MS2) scan with a resolution of 35,000, high collisional dissociation, and a spray voltage of 3.5 kV (positive-ion mode)/−2.5 kV (negative-ion mode) in NCE mode.

### 2.5 Data analysis

#### 2.5.1 Data processing

The raw data from the mass spectrometry downgauge was analyzed with Compound Discoverer 3.2 (Thermo Fisher, United States) for peak processing and peak integration. The measured mass spectral primary and mass spectral secondary information were matched with the mzCloud, Chemspider, and mzVault databases to analyze the metabolites to which they could be matched.

#### 2.5.2 Analysis of differential metabolites

Multivariate statistical analysis was performed using SIMCA 14.1 (Umetrics, Sweden) software. The unsupervised principal component analysis (PCA) was used to categorize the ions detected in the positive and negative modes, and then the data were analyzed using Orthogonal Partial Least Squares Discriminant Analysis (OPLS-DA). The model was verified by the permutation test to find out the projected importance values (VIPs) of the variables. The metabolites were screened for differences between treatment and the control. The metabolite analysis was performed using the online software MetaboAnalyst 5.0. Kyoto Encyclopedia of Genes and Genomes (KEGG) database is a comprehensive database that integrates genomic, chemical, and systemic functional information to reveal the genetic material and chemical blueprint of life phenomena. The samples that met the criteria were compared with the KEGG database to identify the metabolites and explain their biological functions and physicochemical properties. For unidentified compounds, KEGG annotations can be used to determine their identity. Using the MBROLE 2.0 online website, identified differential metabolites were mapped to relevant metabolic pathways to better understand their functions and roles in biological systems.

*p* values were obtained by univariate analysis of the multiplicity of differences (Fold-Change) and T-statistical test, combined with VIP values (Variable Important for the Projection) obtained by multivariate statistical analysis OPLS-DA to screen for differential metabolites. Differential metabolites were required to fulfill the following conditions simultaneously: (1) |Log2 Fold Change| ≥ 1; (2) *P*-value ≤ 0.05; (3) VIP ≥ 1.

## 3 Results

### 3.1 Multivariate statistical analysis

**Figure 1:**
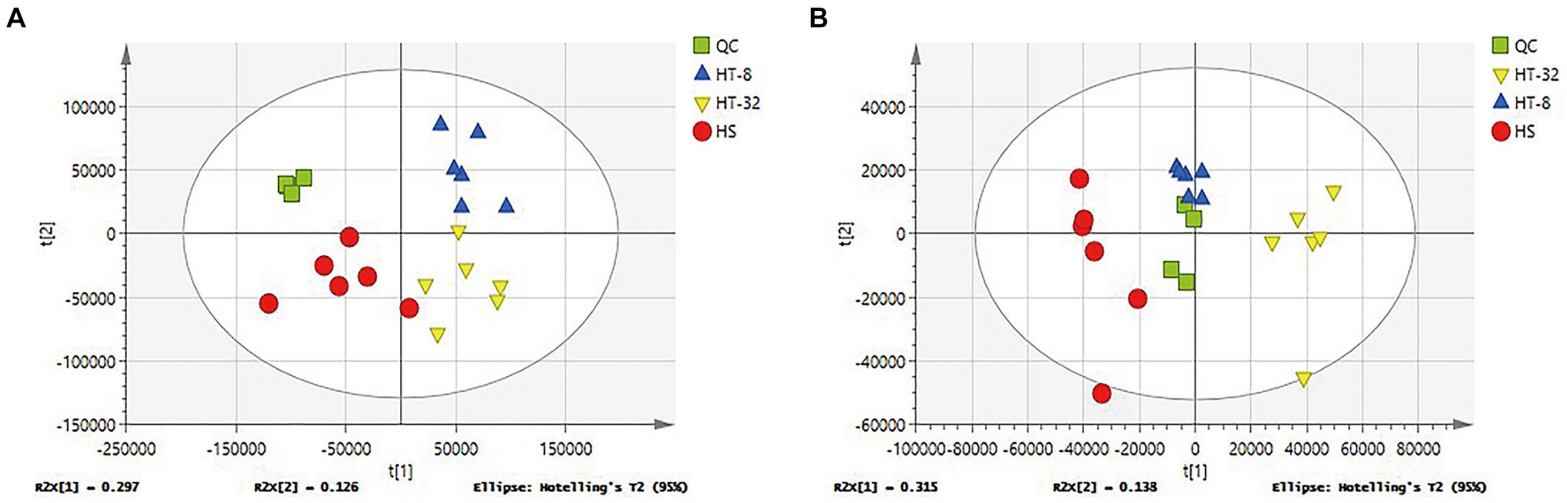
PCA score plots of different samples. **(A)** PCA score plots of three groups in cationic mode (positive ion). **(B)** PCA score plots of three groups in anionic mode (negative ion). Green represents the quality control group (QC), Red represents control group (HS), blue represents 8 mmol/L taurine group (HT-8), yellow represents 32 mmol/L taurine group (HT-32), (*n* = 6) the same as below.

In order to find the differential metabolites between the treatment and control groups, multivariate statistical analysis of cell lysates was performed. The stability of the model was indicated by QC samples. Figure 1 showed the plots of PCA scores for the degree of dispersion between the groups of samples with different concentrations of taurine and the control group in the positive and negative ion mode, respectively. Almost all of the samples were within the ellipse representing the 95% confidence interval, and all treatment groups were separated from the blank group in the horizontal coordinate t [1] (first principal component), and the QC samples were clustered together, which indicated that the established system was stable and reliable. Further, taurine had a significant effect on the metabolism patterns of mammary epithelial cells of cows under high-temperature conditions.

**Figure 2:**
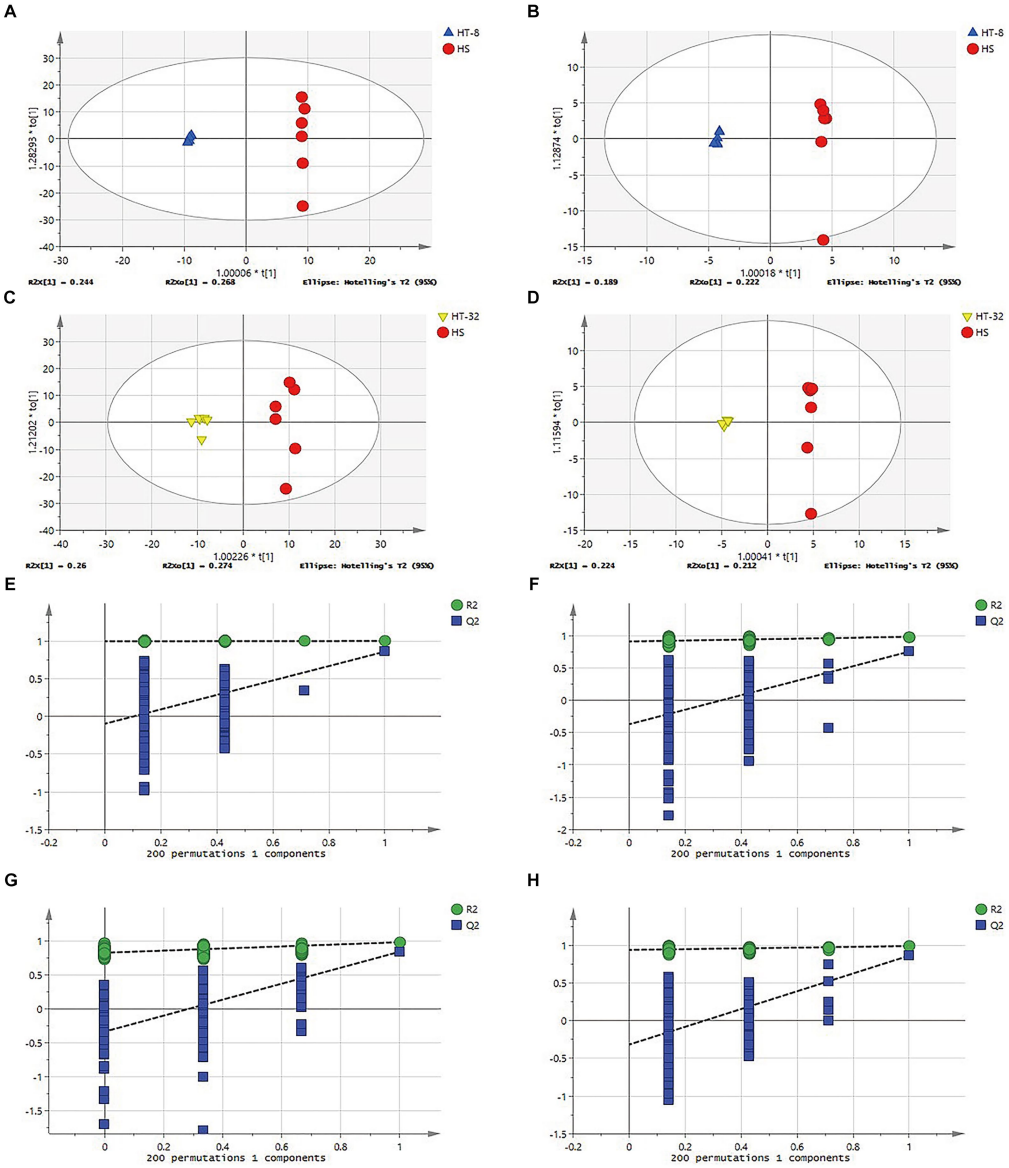
OPLS-DA model and verification diagram of different samples. **(A)**OPLS-DA scores plot of HT-8 vs. HS in cationic mode. **(B)** OPLS-DA scores plot of HT-8 vs. HS in anionic mode. **(C)** OPLS-DA scores plot of HT-32 vs. HS in cationic mode. **(D)** OPLS-DA scores plot of HT-32 vs. HS in anionic mode. **(E–H)** OPLS-DA model verification diagram of panel **(A–D)**.

To verify the reliability of the model and to identify the potential biological markers affecting cellular metabolic patterns, orthogonal partial least squares discriminant analysis (OPLS-DA) was carried out between the HS, HT-8 and HT-32 groups in positive and negative ion modes, respectively. The parameters of the model, R^2^X and R^2^Y, represented the explanatory rate of the constructed model for the X and Y matrices, respectively. The value of Q^2^ indicated the predictive ability of the model, and their sizes directly reflected the reliability of the model. The OPLS-DA score plots between different groups in positive and negative ion mode were shown in Figures 2A–D, and their related model parameters, R^2^ and Q^2^ values, were shown in Table 1. The R^2^ values of each group were greater than 0.5, which showed that the models of the different concentrations of taurine treatment groups were stable; the Q^2^ values of HT-8 and HT-32 groups in positive ion mode were 0.823, 0.836, respectively, and the Q^2^ values of HT-8 and HT-32 group in negative ion mode were 0.780 and 0.863, respectively, indicating that the model predictability was good.

**Table 1:** OPLS-DA model parameters with different concentrations of taurine.

	Positive		Negative	
	HT-8 vs. HS	HT-32 vs. HS	HT-8 vs. HS	HT-32 vs. HS
R^2^X(cum)	0.674	0.534	0.603	0.526
R^2^Y(cum)	0.999	0.976	0.999	0.997
Q^2^(cum)	0.823	0.836	0.780	0.863

In order to decide whether the data derived from the OPLS-DA analysis were analytically significant, the data were subjected to a permutation test (Permutation test). As shown in Figures 2E–H, the slope of the fitted line of the various treatment groups was greater than 0, and the next step in the analysis could be carried out.

### 3.2 Effects of taurine on the metabolite profile of the mammary epithelial cells

**Figure 3:**
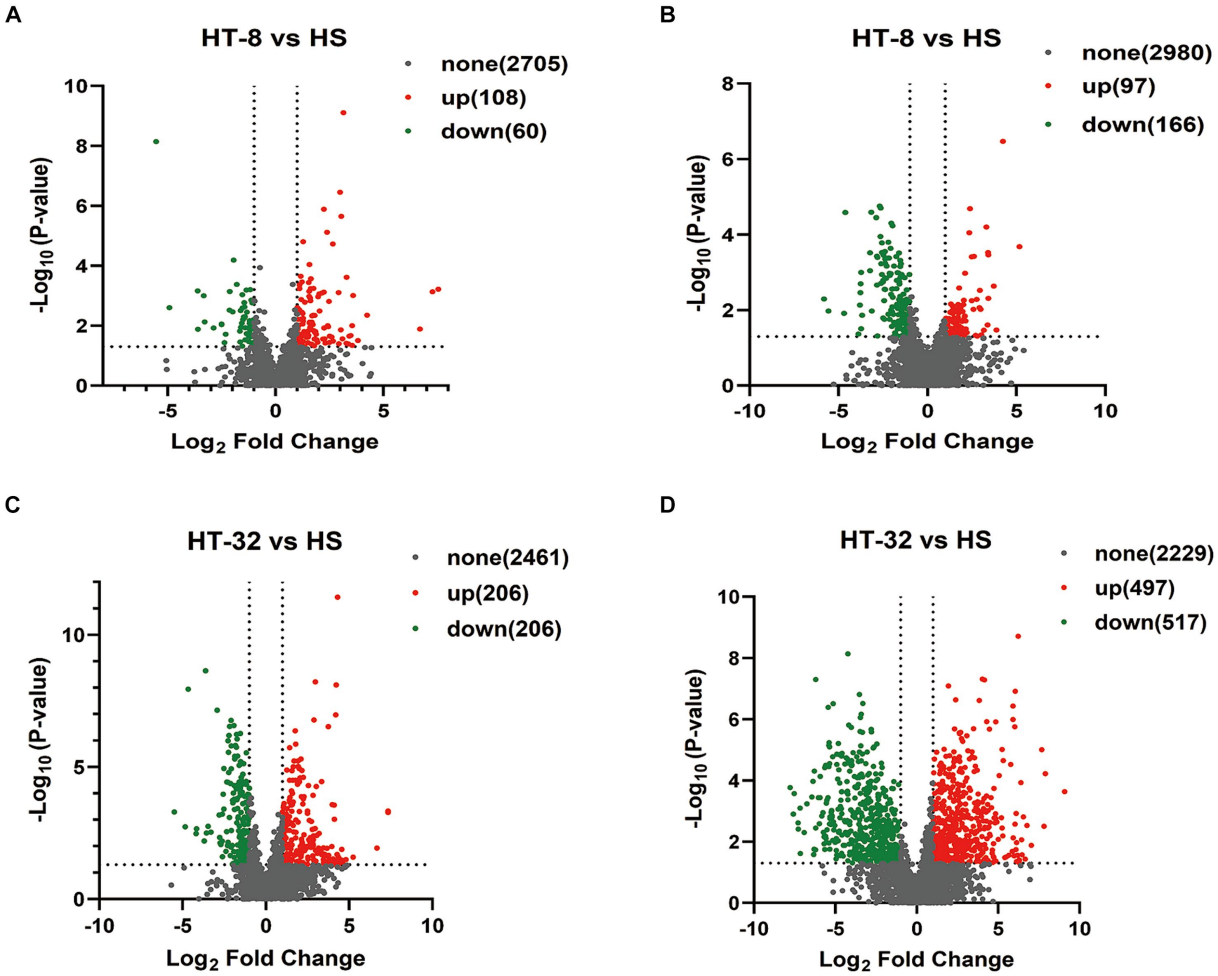
Volcano diagram of differential expressed metabolites between different samples. **(A)** Volcano diagram of HT-8 vs. HS in cationic mode. **(B)** Volcano diagram of HT-8 vs. HS in anionic mode. **(C)** Volcano diagram of HT-32 vs. HS in cationic mode. **(D)** Volcano diagram of HT-32 vs. HS in anionic mode. Red dots represent metabolites with significant and upregulated differences, green dots represent metabolites with significant and downregulated differences, and black represents metabolites with non-significant differences.

Metabolomic analysis was conducted to reveal the changes in metabolic profiles of the mammary epithelial cells following taurine treatment under high temperatures. Compared to the control group, a total of 2,873 metabolites were detected in the positive ion mode when treated with 8 mmol/L taurine (Figure 3A), of which 108 metabolites were significantly up-regulated, 60 metabolites were significantly down-regulated, and the remaining 2,705 metabolites did not show significantly different. On the other hand, a total of 3,243 metabolites were detected in the negative ion mode, among which 97 metabolites were significantly up-regulated, 166 metabolites were significantly down-regulated, and the remaining 2,980 metabolites were not significantly different (Figure 3B). In 32 mmol/L taurine group, 2,873 metabolites were found in the positive ion mode, of which 206 metabolites were significantly up-regulated, 206 metabolites were significantly down-regulated and the other 2,461 metabolites did not show significant changes (Figure 3C). In addition, a total of 3,243 metabolites were detected in negative ion mode, of which 497 metabolites were significantly up-regulated, 517 metabolites were significantly down-regulated, and the remaining 2,229 metabolites showed no significant difference (Figure 3D).

### 3.3 Metabolite clustering analysis

**Figure 4:**
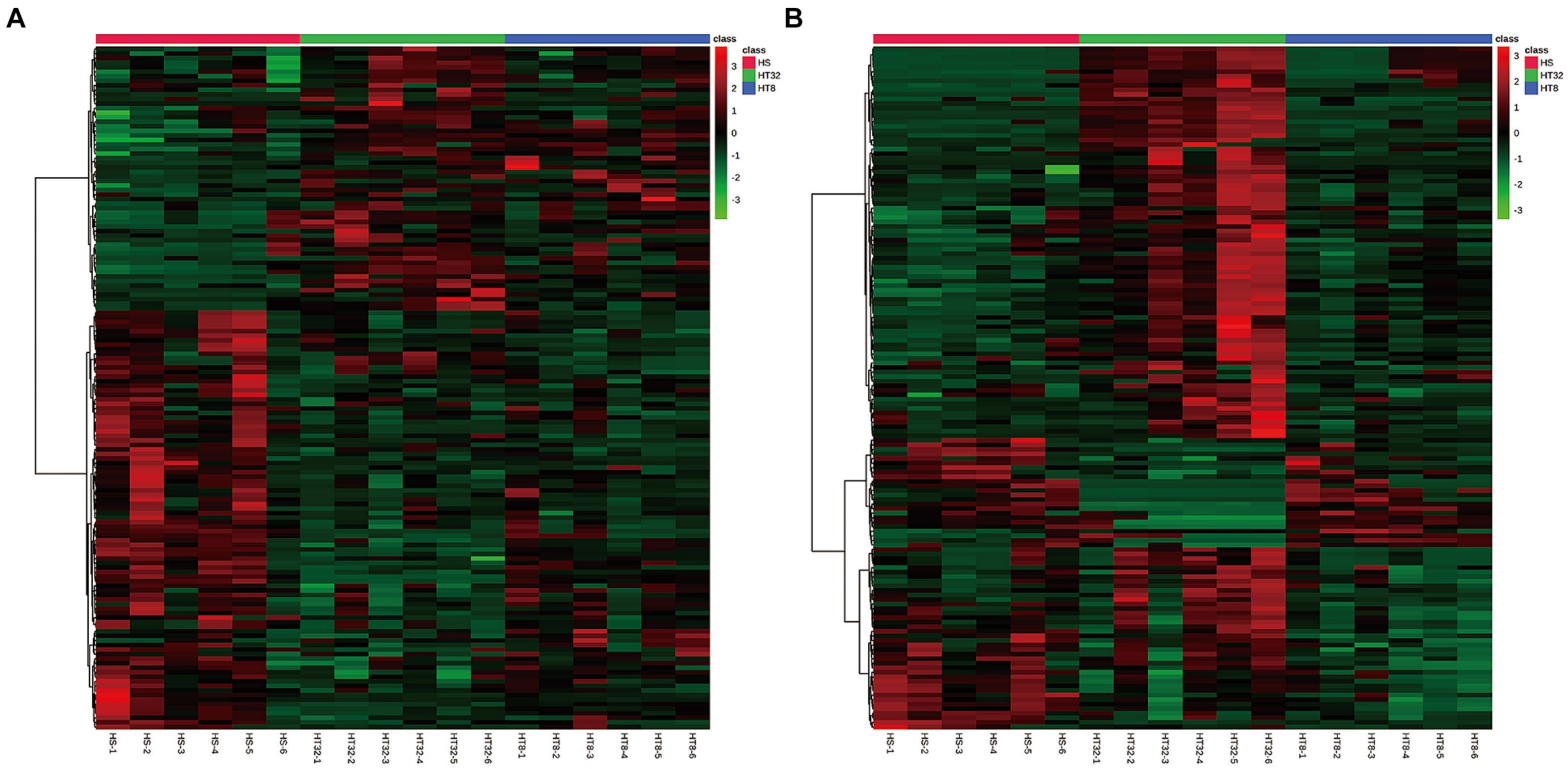
Cluster diagram of differentially expressed metabolites. **(A)** Cluster diagram of three groups in cationic mode (positive ion). **(B)** Cluster diagram of three groups in anionic mode (negative ion). Red dots represent up-regulated metabolites and green dots represent down-regulated metabolites. The vertical axis indicates clustering of all groups and the horizontal axis indicates clustering of all metabolites, with shorter cluster branches indicating higher similarity.

Heatmaps constructed from the peak areas of the differential metabolites showed the combined differentiation between treatments. The clustering of metabolite contents among groups could be clearly observed by horizontal comparison. As shown in Figure 4, the HT-8 and HT-32 groups had similar metabolite expression patterns in the positive ion mode, whereas the HT-8 and HS groups had similar metabolite expression patterns in the negative ion mode.

### 3.4 Analysis of differential metabolites

Based on the OPLS-DA assay, potential metabolic markers were screened using a “multi-criteria” technique. Differential metabolites between treatment and the control group were screened under the conditions of |log2 Fold Change| ≥ 1, VIP value > 1, and *P*-value ≤ 0.05. As shown in Table 2, a total of 15 differential metabolites were screened in the HT-8 group compared with the control group, of which N,N-Dimethyldecylamine N-oxide, Norethindrone, Bis(2-ethylhexyl) phthalate, Taurine, Bacteriopheophytin, L-Glutathione oxidized and PC(18:0/24:0) for seven upregulated metabolites, G Benzylpenicillin, Creatinine, 3-Phosphoglyceric acid, Guanosine 5′-diphosphate, Hypoxanthine, Adenosine 5′-monophosphate, α-D-Glucose-1,6-bisphosphate, Palmitic Acid as 8 downregulated metabolites.

**Table 2:** Differential metabolites of HS vs. HT-8 group.

Name	Molecular formula	Log_2_ FC	*P*-value	VIP	Up/down
N,N-Dimethyldecylamine N-oxide	C12 H27 N O	7.54	0.000609	1.63657	Up
Norethindrone	C20 H26 O2	2.98	0.009498	1.62568	Up
Bis(2-ethylhexyl) phthalate	C24 H38 O4	2.51	0.022253	1.39681	Up
Taurine	C2 H7 N O3 S	2.15	0.000803	2.09867	Up
Bacteriopheophytin	C55 H76 N4 O6	1.7	0.015532	1.34182	Up
L-Glutathione oxidized	C20 H32 N6 O12 S2	1.5	0.036793	1.44644	Up
PC (18:0/24:0)	C50 H100 N O8 P	1.45	0.048642	1.29947	Up
G Benzylpenicillin	C16 H18 N2 O4 S	−1.17	0.041552	1.42848	Down
Creatinine	C4 H7 N3 O	−1.21	0.0084	1.82303	Down
3-Phosphoglyceric acid	C3 H7 O7 P	−1.36	0.042315	1.71386	Down
Guanosine 5′-diphosphate	C10 H15 N5 O11 P2	−1.36	0.044289	1.63198	Down
Hypoxanthine	C5 H4 N4 O	−1.45	0.001688	1.77526	Down
Adenosine 5′-monophosphate	C10 H14 N5 O7 P	−1.66	0.048445	1.23457	Down
α-D-Glucose-1,6-bisphosphate	C6 H14 O12 P2	−1.83	0.039922	1.5497	Down
Palmitic Acid	C16 H32 O2	−4.92	0.002485	1.51669	Down

As shown in Table 3, a total of 30 differential metabolites were screened in the HT-32 group compared with the control group, including 13 upregulated metabolites such as Phenol, Norethindrone, Nonanoic acid, Decanoic acid, and Guanine, and ADP, L-Glutathione (reduced), UDP, GDP, GTP, ATP, Hypoxanthine, Phytosphingosine, 2-Amino-1,3-octadecanediol, Palmitic Acid and 17 other downregulated metabolites.

**Table 3:** Differential metabolites of HS vs. HT-32 group.

Name	Molecular formula	Log_2_ FC	*P*-value	VIP	Up/down
Phenol	C6 H6 O	7.89	5.97E−05	1.13794	Up
N,N-Dimethyldecylamine N-oxide	C12 H27 N O	7.34	0.000522	1.18492	Up
Taurine	C2 H7 N O3 S	6.05	1.21E−07	1.58637	Up
Norethindrone	C20 H26 O2	6.02	1.75E−06	1.19654	Up
Nonanoic acid	C9 H18 O2	4.55	0.000363	1.39485	Up
Decanoic acid	C10 H20 O2	3.49	2.02E−06	1.39083	Up
Bacteriopheophytin	C55 H76 N4 O6	3.07	3.48E−06	1.48380	Up
PC (18:0/24:0)	C50 H100 N O8 P	2.6	0.004076	1.09031	Up
Adipic acid	C6 H10 O4	1.99	0.037432	1.14083	Up
Valeric acid	C5 H10 O2	1.45	4.69E−05	1.41050	Up
Caprylic acid	C8 H16 O2	1.33	0.001663	1.39478	Up
Mangostin	C24 H26 O6	1.18	0.000708	1.29905	Up
Guanine	C5 H5 N5 O	1.09	0.000243	1.22641	Up
Adenosine diphosphate (ADP)	C10 H15 N5 O10 P2	−1	0.039933	1.13877	Down
chloroacetic acid	C2 H2 Cl2 O2	−1	0.000828	1.44780	Down
L-Histidine	C6 H9 N3 O2	−1.11	0.014266	1.58664	Down
Phosphoenolpyruvic acid	C3 H5 O6 P	−1.13	0.038488	1.23543	Down
Isoniazid	C6 H7 N3 O	−1.14	0.036489	0.90460	Down
DL-Arginine	C6 H14 N4 O2	−1.2	0.036092	1.36733	Down
L-Glutathione (reduced)	C10 H17 N3 O6 s	−1.25	0.026896	1.29385	Down
Uridine 5′-diphosphate (UDP)	C9 H14 N2 O12 P2	−1.35	0.0296	1.30708	Down
Guanosine 5′-diphosphate (GDP)	C10 H15 N5 O11 P2	−1.41	0.018383	1.25923	Down
Guanosine triphosphate (GTP)	C10 H16 N5 O14 P3	−1.43	0.006164	1.16313	Down
Adenosine triphosphate (ATP)	C10 H16 N5 O13 P3	−1.45	0.004106	1.10366	Down
2,3-Bisphospho-D-glyceric acid	C3 H8 O10 P2	−1.49	0.007239	1.27946	Down
Creatinine	C4 H7 N3 O	−1.75	4.95E−05	1.07949	Down
Hypoxanthine	C5 H4 N4 O	−1.98	0.000226	1.65350	Down
Phytosphingosine	C18 H39 N O3	−2.4	0.015633	1.25813	Down
2-Amino-1,3-octadecanediol	C18 H39 N O2	−4.85	0.001825	1.23619	Down
Palmitic acid	C16 H32 O2	−5.5	0.000499	1.75619	Down

**Figure 5:**
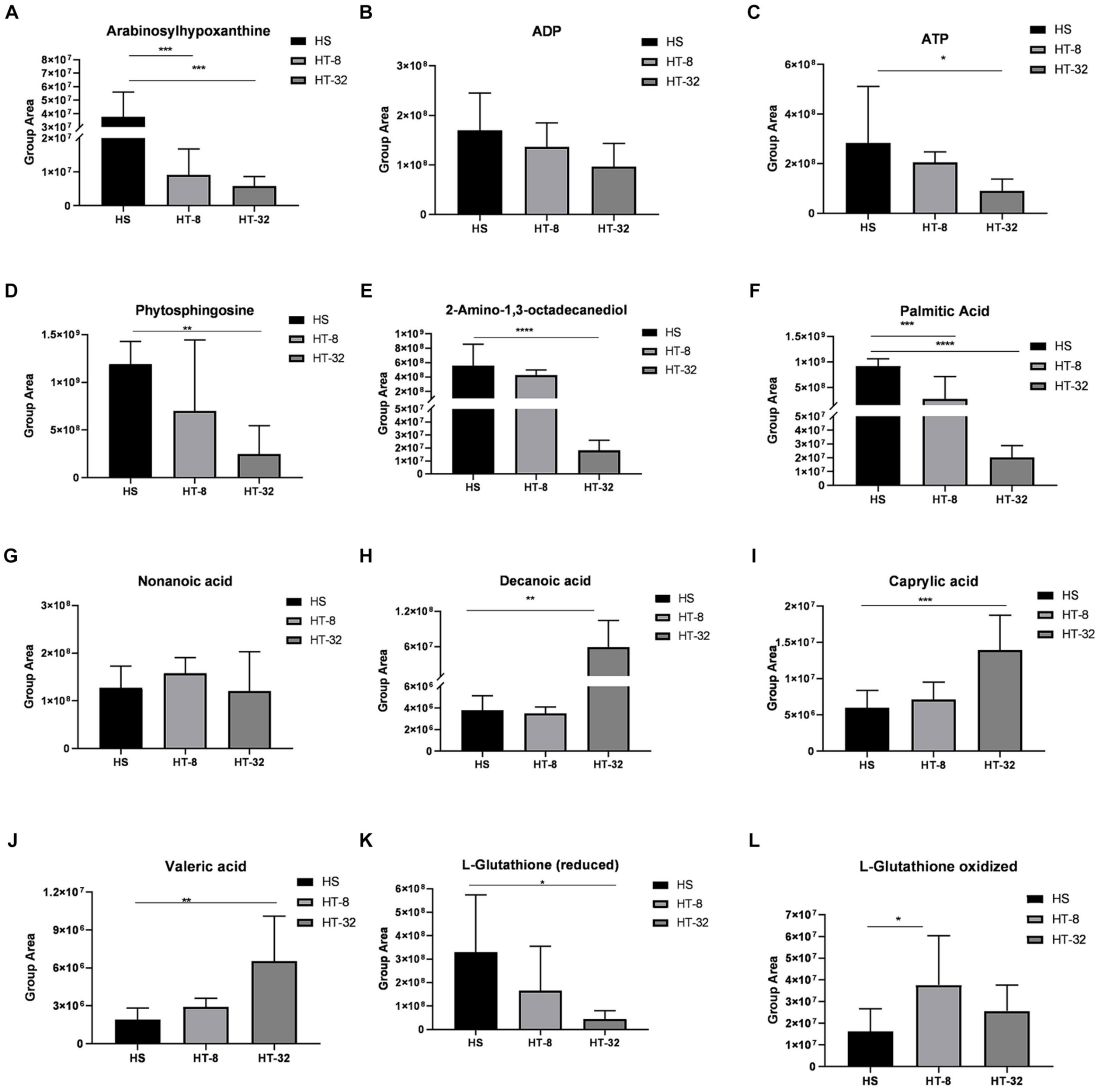
A plot of the peak values of several major metabolites between treatments. (**A-L**) indicate peak plots of Arabinosylhypoxanthine, ADP, ATP, Phytosphingosine, 2-Amino-1,3-octadecanediol, Palmitic acid, Nonanoic acid, Decanoic acid, Caprylic acid, Valeric acid, L-Glutathione (reduced) and L-Glutathione (oxidized) between treatments, respectively. Values are means ± SEM (*n* = 3). Asterisks (*, ** and ***) represent significant differences with *p* < 0.05, *p* < 0.01 and *p* < 0.001 respectively.

Further analysis of these differential metabolites, as shown in Figure 5, revealed that 8 mmol/L and 32 mmol/L taurine resulted in the upregulation of decanoic acid, valeric acid, octanoic acid, and oxidized glutathione, and the downregulation of ADP, ATP, hypoxanthine, phytosphingosine, 2-amino-1,3-octadecanediol (sphingolipid analogs), reduced glutathione, and palmitic acid, compared to the control.

### 3.5 Analysis of differential metabolite metabolic pathways

**Figure 6:**
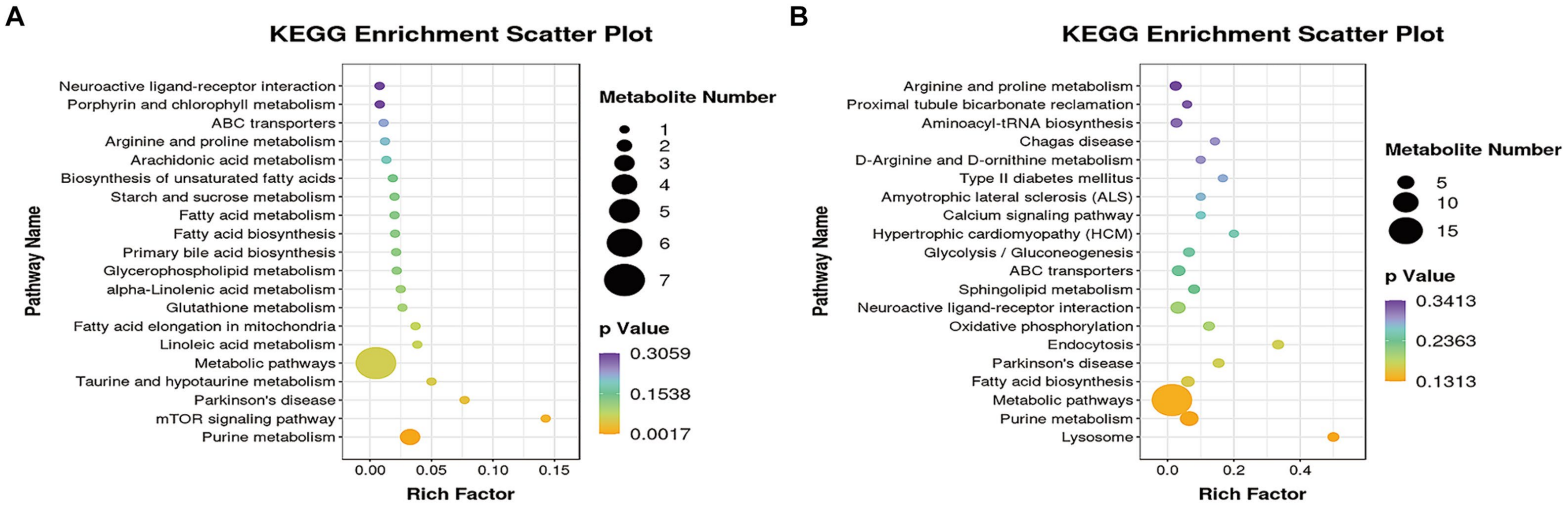
The KEGG pathway analysis of differentially expressed metabolites. **(A)** The KEGG pathway of HT-8 vs. HS. **(B)** The KEGG pathway of HT-32 vs. HS. The size of the solid circle in the figure indicated the number of differential metabolites enriched by the pathway, and the darkness of the color of the circle represents the size of the *p*-value.

In order to identify potential biomarkers of KEGG identification, the online software of MetaboAnalyst 5.0 was utilized to analyze KEGG. KEGG pathway enrichment analysis was performed by the MBROLE.2.0·online website. Figure 6 showed differentially expressed metabolites through the KEGG pathway analysis. Figures 6A,B showed the top 20 KEGG metabolic pathways enriched by differential metabolites between the HT-8 vs. HS and HT-32 vs. HS groups, respectively. After being treated with 8 mmol/L taurine, purine metabolism, glutathione metabolism, fatty acid biosynthesis and metabolism, linoleic acid metabolism, and mTOR signaling pathway were significantly changed (Figure 6A). The changes in purine metabolism, fatty acid metabolism, taurine and hypo-taurine metabolism, mTOR signaling pathway, and metabolic pathway were significant after 32 mmol/L taurine (HT-32) treatment (Figure 6B). In conclusion, taurine affected the metabolic pathways: purine metabolism, amino acid metabolism, glucose metabolism, fatty acid metabolism and biosynthesis in bovine mammary epithelial cells under high temperature condition.

## 4 Discussion

The aim of this study was to assess the impact of taurine supplementation on the metabolite composition of bovine mammary epithelial cells subjected to high-temperature stress. In this experiment, employing liquid chromatography-mass spectrometry (LC-MS), a total of 15 differential metabolites were identified in the mammary epithelial cells of dairy cows by supplementation of 8 mmol/L taurine under high temperature, and 30 differential metabolites were identified with the addition of 32 mmol/L taurine, compared with the control group. The KEGG enrichment analysis revealed that these metabolites mainly regulated the pathways of purine metabolism, fatty acid metabolism, fatty acid biosynthesis and sphingolipid metabolism.

Fatty acids and their derivatives are widespread in all living organisms, both as energy providers and as part of the composition of biological membranes (35). They can regulate gene expression via activating transcription factors and cell membrane receptor signaling (36). They also affect their own metabolism as well as the metabolism of other compounds by influencing gene transcription (37). Under high temperature condition, cells need to regulate membrane fluidity to maintain normal physiological function, as the amount of saturated fatty acids in the membrane affects the fluid properties of the membrane. Therefore, cells increase the synthesis of unsaturated fatty acids to regulate membrane properties in response to HS (38). The synthesis of short- and medium-chain unsaturated fatty acids are fatty acid groups consisting of a carbon chain of 3–12 carbon atoms, which have a small molecular mass and a strong ability to penetrate cell membranes. Short-chain fatty acids include formic acid, acetic acid, and valeric acid, while medium-chain fatty acids include caproic acid, octanoic acid, nonanoic acid, decanoic acid, etc. (39, 40). The medium chain fatty acids include hexanoic acid, octanoic acid, nonanoic acid and decanoic acid. Studies have shown that pathways related to lipid metabolisms, such as fatty acid biosynthesis and amino acid metabolism, are inhibited under high temperature conditions (41). In the present study, 32 mmol/L taurine significantly upregulated the expression of short-chain fatty acids, such as caproic acid, valeric acid, nonanoic acid, and decanoic acid in the mammary epithelial cells of dairy cows under high temperature conditions. Therefore, it can be hypothesized that taurine could protect the cells by regulating the expression of short and medium chain fatty acids in the fatty acid synthesis pathway, thereby increasing the expression of heat shock proteins and enhancing the tolerance to heat stress.

Palmitic acid (PA) is the most abundant saturated free fatty acid in the blood and one of the main substrates for fat synthesis (42). PA can regulate cellular metabolism via the activation of phosphatidylinositol 3-kinase (PI3K) (43). Excessive PA leads to endoplasmic reticulum stress, mitochondrial damage, and apoptosis (44–46). Excessive PA also induces programmed necrosis (necrotic apoptosis) in endothelial cells by initiating enhanced autophagy (47). Besides, PA has been reported to stimulate hepatocyte apoptosis by inducing oxidative stress through intracellular generation of reactive oxygen species (48) and apoptosis of bovine mammary epithelial cells by inducing extreme endoplasmic reticulum stress (49). In this study, we found that 8 mmol/L and 32 mmol/L of taurine downregulated the expression of palmitic acid in bovine mammary epithelial cells under high temperature conditions. Therefore, taurine may activate cellular autophagy pathways, and alleviate endoplasmic reticulum stress, mitochondrial damage, and apoptosis induced by palmitic acid. Moreover, taurine can reduce cellular autophagy by inhibiting the PI3K pathway and inhibiting the mTOR pathway through down-regulation of adenosine monophosphate (AMP) expression. It also reduces the production of intracellular reactive oxygen species under high temperature conditions, thereby alleviating oxidative stress and to some extent relieving the damage of mammary epithelial cells in dairy cows under high temperature conditions.

Sphingolipids are one of the major lipid classes in eukaryotes and play a crucial role in cells as structural components of membrane lipid bilayers and signaling molecules (50, 51). They are related to important physiological and pathological processes, including cell adhesion, recognition of viral and bacterial toxins, skin barrier formation, neural function, apoptosis, and glucose metabolism (52, 53). Phytosphingosine (PHS) is a sphingolipid found in plants and animals and is unique since it has an extra hydroxyl group compared to other long-chain bases (54). 2-Amino-1,3-octadecanediol (2-Amino-1,3-octadecanediol), a sphingosine analog that inhibits protein kinase C, is used in the treatment of skin diseases and cancer (55). Studies have shown that sphingolipid metabolites associated with PHS, as well as sphingosine 1-phosphate, can cause programmed cell death (56). They can also directly or indirectly interfere with mitochondria and induce apoptosis (57) and promote apoptosis by disrupting mitochondrial autophagy (54, 58). Furthermore, PHS causes abnormal nuclear morphology, micronuclei and DNA damage, inhibits cell proliferation by damaging DNA, and activates the ATM/P53/p21 pathway, resulting in cell cycle arrest in S phase (59). In this experiment, we found that the metabolites of PHS and its analog (2-Amino-1,3-octadecanediol) in the mammary epithelial cells of dairy cows under high temperature conditions were significantly downregulated by 32 mmol/L taurine. It means that taurine might alleviate apoptosis under high temperature conditions by down-regulating sphingomyelin metabolism.

Purine nucleotides, essential components for cell proliferation, provide cells with energy, and help cells against the adverse effects of the external environment (60, 61). When an animal is subjected to HS, it will release some endogenous substances, such as catalase and cytokines, which can affect the purine metabolic pathway and lead to changes in the activity of the relevant enzymes (62). Heat stress may also lead to a change in purine metabolism to produce excessive free radicals and oxidative stress, which may adversely affect the cells (63). Hypoxanthine is an important product of the nucleotide degradation pathway and can be used as a substrate for ATP synthesis (64, 65). It can induce apoptosis by regulating the expression of proteins associated with apoptosis (66), which induces cell death and ROS production. Studies have shown that HS first affects the animal’s feed intake and nutrient absorption, and then affects the body’s metabolism. During heat production, a large number of free radicals are generated, causing oxidative stress in the body (67, 68). In this experiment, 8 and 32 mmol/L taurine inhibited purine metabolism by significantly down-regulating the production of ATP, ADP, UTP, UDP and hypoxanthine in mammary epithelial cells of cows at high temperature. By reducing the levels of these metabolites, taurine attenuates high temperature-induced oxidative stress and free radical release. Thus, it attenuates the damage and apoptosis of mammary epithelial cells in dairy cows under high temperature conditions.

The results of this study emphasize the vital role of taurine in alleviating HS in dairy cows through various metabolic pathways. These effects not only help maintain the health and productivity of dairy herds but also have substantial implications for improving the economic sustainability of dairy farming operations. Therefore, ensuring sufficient taurine intake through careful nutritional management and feeding practices is essential for managing HS and optimizing overall herd health and performance.

In summary, the effect of taurine on the metabolome of mammary epithelial cells of dairy cows under high temperature conditions is a complicated biological process, and the potential biomarkers are mainly involved in the regulation of purine metabolism, lipid metabolism, sphingolipid metabolism, amino acid metabolism, which constitutes a complicated regulatory network. However, this study primarily utilized metabolomics analysis to pinpoint potential biomarkers indicating taurine’s capacity to mitigate HS, it did not proceed to validate the precise mechanisms underlying the action of the selected metabolites. To strengthen the findings, future research could incorporate cellular function experiments, such as assessing cell proliferation and apoptosis, to validate the metabolomics results and delve further into taurine’s specific effects on mammary epithelial cells.

## 5 Conclusion

The LC-MS technique and multivariate statistical analysis were utilized to screen the significantly different metabolites of the mammary epithelial cells of dairy cows under high-temperature conditions after taurine treatment with different concentrations of taurine. These metabolites are mainly involved in the pathways of purine metabolism, lipid metabolism, and sphingomyelin metabolism, which accumulates data for the in-depth study of the mitigation of heat stress in dairy cows by taurine.

## Data availability statement

The original contributions presented in the study are included in the article/supplementary material, further inquiries can be directed to the corresponding author.

## Ethics statement

The animal study was approved by the Ethics Committee/Institutional Review Board. The study was conducted in accordance with the local legislation and institutional requirements.

## Author contributions

FL: Data curation, Writing – original draft, Formal analysis, Methodology. LL: Methodology, Writing – review & editing, Visualization. ZL: Writing – review & editing, Data curation. GZ: Writing – review & editing. FZ: Methodology, Writing – review & editing. LW: Funding acquisition, Writing – review & editing, Supervision.
